# An Interaction of Rhamnolipids with Cu^2+^ Ions

**DOI:** 10.3390/molecules23020488

**Published:** 2018-02-23

**Authors:** Jolanta Cieśla, Magdalena Koczańska, Andrzej Bieganowski

**Affiliations:** Institute of Agrophysics, Polish Academy of Sciences, Doświadczalna 4, 20-290 Lublin, Poland; m.koczanska@ipan.lublin.pl (M.K.); a.bieganowski@ipan.lublin.pl (A.B.)

**Keywords:** rhamnolipids, biosurfactants, micellization, metal complexation

## Abstract

This study was focused on the description of interaction between Cu^2+^ ions and the 1:1 mono- and dirhamnolipid mixtures in the premicellar and aggregated state in water and 20 mM KCl solution at pH 5.5 and 6.0. The critical micelle concentration of biosurfactants was determined conductometrically and by the pH measurements. Hydrodynamic diameter and electrophoretic mobility were determined in micellar solutions using dynamic light scattering and laser Doppler electrophoresis, respectively. The copper immobilization by rhamnolipids, methylglycinediacetic acid (MGDA), and ethylenediaminetetraacetic acid (EDTA) was estimated potentiometrically for the Cu^2+^ to chelating agent molar ratio from 16:100 to 200:100. The degree of ion binding and the complex stability constant were calculated at a 1:1 metal to chelant molar ratio. The aggregates of rhamnolipids (diameter of 43–89 nm) were negatively charged. Biosurfactants revealed the best chelating activities in premicellar solutions. For all chelants studied the degree of metal binding decreased with the increasing concentration of the systems. The presence of K^+^ lowered Cu^2+^ binding by rhamnolipids, but did not modify the complex stability significantly. Immobilization of Cu^2+^ by biosurfactants did not cause such an increase of acidification as that observed in MGDA and EDTA solutions. Rhamnolipids, even in the aggregated form, can be an alternative for the classic chelating agents.

## 1. Introduction

Rhamnolipids are the anionic biosurfactants which are synthesized by *Pseudomonas aeruginosa*. Their molecules are composed of one or two rhamnose units glycosidically linked with the beta-hydroxydecanoic acid units [1]. The acidic character of rhamnolipids results from the presence of carboxylic groups (p*K*_a_ 5.6) in their molecules [1,2]. Due to the low toxicity, high biodegradability, and emulsifying activity [1,3] they are promising substances which can replace the synthetic surfactants. The possible areas of their application include, e.g., food production and protection, the pharmaceutical and cosmetic industry, agriculture, and natural environment protection [4,5]. Affinity of rhamnolipids to metals is studied mostly in a context of remediation of the soil contaminated with heavy metals [6,7,8,9]. However, some of divalent metals (e.g., Cu, Zn), which show toxicity to live organisms at high concentration, belong simultaneously to the microelements, which are necessary for proper functioning of these organisms. Such micronutrients are often supplemented in the chelated form.

Ethylenediaminetetraacetic acid (EDTA) is probably the most popular chelating agent belonging to aminopolycarboxylates. It is used in cosmetics and pharmaceuticals without the toxic or harmful effects on human health [10]. However, its complexes with metals are very stable and by the persisting in the environment cause the perturbations of natural metal speciation. Moreover, it was shown by Sillanpää and Oikari [11] that EDTA, in the form of chelate with metal, is much more toxic than in the free state. Low biodegradability of aminopolycarboxylates resulted in looking for the other more environmentally friendly components.

Methylglycinediacetic acid (MGDA) is a complexing agent belonging to a new generation of biodegradable chelants [12,13,14]. It forms water-soluble complexes with polyvalent ions, but is degraded in the natural environment two times faster than EDTA [13]. Considering the above, MGDA is a greener alternative to EDTA [15].

**Figure 1:**
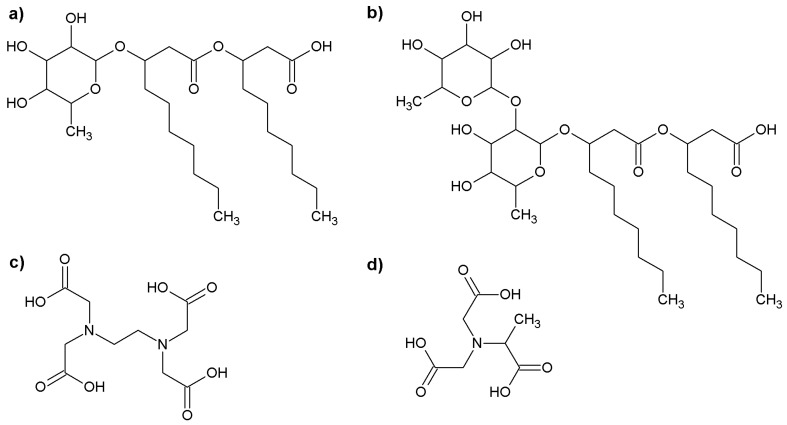
The structures of (**a**) monorhamnolipid; (**b**) dirhamnolipid; (**c**) ethylenediaminetetraacetic acid (EDTA); and (**d**) methylglycinediacetic acid (MGDA).

The chemical structures of the above discussed compounds are shown in Figure 1.

There is an observed increase in interest in the application of natural, biodegradable, and effective compounds in different areas of industry to replace synthetic ones, which have been used so far. Rhamnolipids, due to their both surface activity and metals mobilization could be compounds which play the role of emulsifying and complexing agent, simultaneously, in different formulations. Therefore, the aim of research was to characterize the interaction between Cu^2+^ ions and the 1:1 mono- and dirhamnolipids mixture in premicellar and aggregated state in water and 20 mM KCl solution at pH 5.5 and 6.0, and to compare it with that of MGDA and EDTA. The above mentioned pH values were chosen as the most often applied and appropriate for both the plant care [16] and cosmetic product [17] formulations.

## 2. Results and Discussion

### 2.1. The Aggregation of the Rhamnolipid Molecules

**Figure 2:**
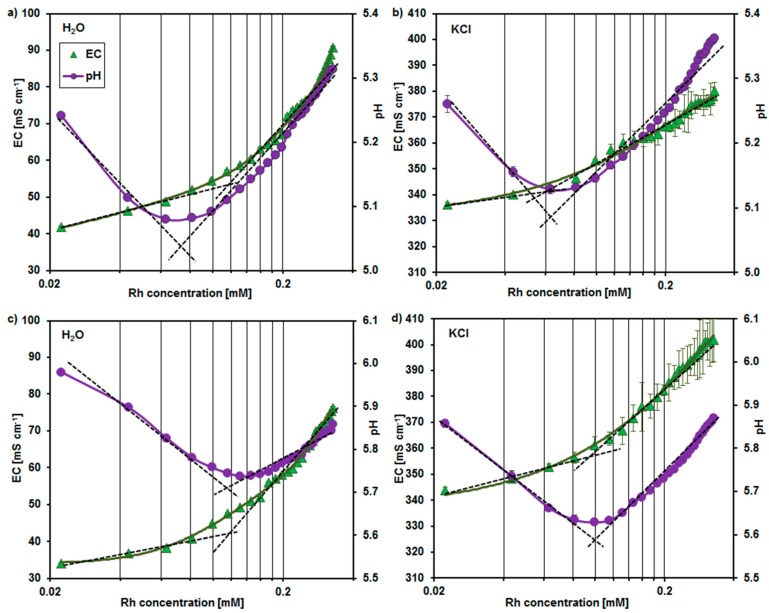
Dependence of electrolytic conductivity (EC) and pH on the rhamnolipids (Rh) concentration in water (**a**,**c**) and KCl solution (**b**,**d**) at pH 5.5 (**a**,**b**) and 6.0 (**c**,**d**); bars represent standard deviation.

In the first step of investigations the critical micelle concentration (CMC) of rhamnolipids was determined at pH 5.5 and 6.0. It was based on the analysis of the pH and electrolytic conductivity (EC) changes with an increasing rhamnolipid concentration in both the water and 20 mM KCl solution. The obtained results are shown in Figure 2.

In the premicellar solutions the EC increased whereas pH decreased with an increasing rhamnolipid content. It was due to the acidic character of studied biosurfactants. The rising amount of rhamnolipids in solution resulted in an increasing concentration of the hydrated H^+^ ions and led to the EC enhancing.

In the micellar solutions the rate of EC increase with the increasing biosurfactant content was slowed down by the process of rhamnolipid aggregation. It is known that the dissociation degree of surfactants in micellar form is lower than this of their free molecules in bulk solution [18]. As a consequence, at the rhamnolipid concentration higher than CMC the acidity of solutions decreased with the increasing amount of surfactants, and pH value tended to that of the stock solution (i.e., pH 5.5 or 6.0).

Determination of the break points of the presented in Figure 2 curves allowed to obtain the CMC values for the rhamnolipid solutions with initial pH of 5.5 and 6.0, respectively. The results are summarized in Table 1.

It can be seen that the CMC was lowered with the increasing ionic strength and the decreasing pH of solution. Moreover, the CMC values, which were estimated on the base of EC analysis, were comparable with these coming from the studies of pH changes. This suggests that the pH measurements can be useful for indication of the CMC of acidic surfactants. To our knowledge such method of CMC determination has not been used so far.

The results which were obtained for the 1:1 mixture of mono- and dirhamnolipids correspond to the literature data. The CMC of dirhamnolipids which was determined by Sánchez et al. [19] on the base of the surface tension measurements and the isothermal titration calorimetry was 0.010 mM and 0.110 mM at pH 4.0 and 7.4, respectively. Dirhamnolipids, which were analyzed by Özdemir et al. [20], aggregated at concentrations of 0.040 mM and 0.150 mM at pH 5.0 and 6.8, respectively. The CMC of monorhamnolipids, which was determined using conductometry, was equal to 0.040 mM at pH 5.0 and 0.100 mM at pH 6.8 of pure aqueous solution [20]. In the 50 mM NaCl solution at pH 6.8 the monorhamnolipids’ CMC was equal to 0.100 mM, whereas the CMC of dirhamnolipids was equal to 0.080 mM [21].

### 2.2. The Complexation of Cu^2+^ Ions by Rhamnolipids

**Figure 3:**
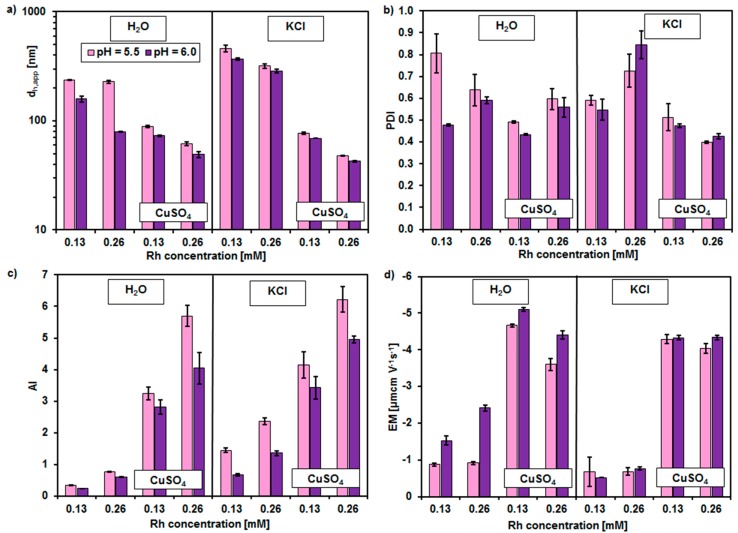
(**a**) Hydrodynamic diameter (d_h,app_); (**b**) polydispersity index (PDI); (**c**) aggregation index (AI); and (**d**) electrophoretic mobility (EM), which were determined in micellar solutions of rhamnolipids (Rh) in water and 20 mM KCl at pH 5.5 and 6.0 without and with the presence of Cu^2+^ ions (at the 1:1 Cu^2+^ to rhamnolipid molar ratio).

The micellar solutions of rhamnolipids with and without the presence of Cu^2+^ ions were characterized in respect to the particles size (d_h,app_), polydispersity (PDI), aggregation (AI), and electrophoretic mobility (EM). The results are shown in Figure 3.

For all the systems analyzed the increase of rhamnolipid concentration from 0.13 to 0.26 mM resulted in a decrease of hydrodynamic diameter of micelles (Figure 3a). It was from 237 ± 2.2 nm to 227.5 ± 7.1 nm at pH 5.5 and from 158.6 ± 9.7 nm to 79.7 ± 1 nm at pH 6.0 in pure micellar solution. The addition of Cu^2+^ ions resulted in decrease of particles size but the lowering of hydrodynamic diameter with the increasing content of rhamnolipids was still visible. When the biosurfactants concentration changed from 0.13 mM to 0.26 mM at pH 5.5 the aggregates diameter decreased from 88.9 ± 1.7 nm to 61.6 ± 2.6 nm, whereas at pH 6.0 it decreased from 73.1 ± 1.1 nm to 49.1 ± 3.5 nm. In the 20 mM KCl solution similar effect of biosurfactants concentration was observed. Without the Cu^2+^ ions added the particles size decreased from 461 ± 34.9 nm to 317.8 ± 13.8 nm and from 367.9 ± 7.1 to 287 ± 11.2 nm at pH 5.5 and 6.0, respectively. In a presence of Cu^2+^ ions in this system the decrease was from 77.1 ± 1.8 nm to 47.9 ± 0.3 nm at pH 5.5 and from 69.3 ± 0.2 nm to 42.6 ± 0.7 nm at pH 6.0. At the given concentration of rhamnolipids the particles size decreased with both the pH and the ionic strength increase.

The observed decrease of particles size with the increasing concentration of biosurfactants was probably a consequence of electrostatic repulsion between the aggregates. Similar effect was found for the synthetic anionic surfactant sodium dodecyl sulfate (SDS) [22]. An enhancement of the ionic strength of dispersing medium over the 1 M can eliminate the influence of electrostatic interaction on the particles size determined by dynamic light scattering (DLS) method in the micellar solution of ionic surfactants [23]. The change of pH affect the morphology of rhamnolipid aggregates [24,25]. It was observed by Dahrazma et al. [24] that in acidic conditions the vesicles with a radius about 55–60 nm whereas, in more alkaline conditions, the small micelles were formed by the rhamnolipids.

The rhamnolipids’ micellar solutions were characterized by different values of PDI (Figure 3b). Generally, the polydispersity ranged from 0.5 to 0.8 in the pure micellar solutions. In the presence of Cu^2+^ ions the PDI values equaled 0.4–0.6 and 0.4–0.5, in the systems without and with KCl, respectively. An increase of pH did not affect PDI significantly, in exception of the aqueous systems at the lowest concentration of rhamnolipids (0.13 mM), where PDI decreased with an increasing alkalization.

The index of aggregation (Figure 3c) increased with the biosurfactants content in all analyzed systems. In the pure aqueous solution AI increased from 0.3 ± 0.1 to 0.8 ± 0.1 at pH 5.5 and from 0.2 ± 0.1 to 0.6 ± 0.1 at pH 6.0. In the presence of KCl the increase was from 1.4 ± 0.1 to 2.4 ± 0.1 at pH 5.5 and from 0.7 ± 0.1 to 1.4 ± 0.1 at pH 6.0. In the aqueous systems containing Cu^2+^ ions the AI changed from 3.2 ± 0.2 to 5.7 ± 0.3 at pH 5.5 and from 2.8 ± 0.2 to 4.0 ± 0.5 at pH 6.0. In the systems with KCl and CuSO_4_ the AI increased from 4.1 ± 0.4 to 6.2 ± 0.4 at pH 5.5 and from 3.4 ± 0.4 to 5.0 ± 0.1 at pH 6.0 when the rhamnolipids content changed from 0.13 mM to 0.26 mM. In all cases, the lower the pH was, the higher the AI values were. It was connected with the effect of pH on the micellization process. At a given concentration of rhamnolipids their molecules were more aggregated at a lower pH (see Table 1). The addition of both KCl and CuSO_4_ resulted in higher values of AI than these in the systems without the salts. It is consistent with the literature data. It is known, that in a presence of electrolyte the aggregates of ionic surfactants are composed of higher number of monomers than those in the pure aqueous systems [18].

The electrophoretic mobility of rhamnolipid aggregates was characterized by the negative sign (Figure 3d), what was a consequence of dissociation of carboxylic groups in the micelle hydrophilic surface/bulk solution interface [1]. The concentration of biosurfactants influenced the EM values differently. At pH 5.5, in both the pure aqueous solution and 20 mM KCl solution, EM was on the level of −0.9 µm cm V^−1^ s^−1^ and −0.7 µm cm V^−1^ s^−1^, respectively, and was independent from the rhamnolipid content. At pH 6.0 in pure aqueous solution the EM changed from −1.5 ± 0.1 µm cm V^−1^ s^−1^ to −2.4 ± 0.1 µm cm V^−1^ s^−1^ whereas, in the presence of KCl, it was from −0.5 ± 0.1 µm cm V^−1^ s^−1^ to −0.8 ± 0.1 µm cm V^−1^ s^−1^ with the rhamnolipid concentration increasing from 0.13 mM to 0.26 mM. This increase of the absolute value of EM was connected with a decrease of the particles size (Figure 3a). In a presence of Cu^2+^ ions in aqueous solutions the absolute value of EM decreased with an increasing content of biosurfactants. It was from 4.7 ± 0.1 µm cm V^–1^ s^–1^ to 3.6 ± 0.2 µm cm V^–1^ s^–1^ and 5.1 ± 0.1 µm cm V^−1^ s^–1^ to 4.4 ± 0.1 µm cm V^–1^ s^−1^ at pH 5.5 and 6.0, respectively. The presence of Cu^2+^ ions in the systems containing KCl reduced the influence of the rhamnolipid concentration on EM. At both pH 5.5 and 6.0 the EM value was about −4.3 ± 0.1 µm cm V^–1^ s^–1^. Generally, the absolute value of EM increased with the pH increase. It resulted from the increase of the amount of ionized biosurfactant molecules. The adjustment of the ionic strength by KCl eliminated this effect. The increase of the absolute value of EM after the addition of Cu^2+^ can be confusing. It is known that the electrical double layer is more compact in the presence of electrolyte, so these values should be lower than in the systems without CuSO_4_. However, it can be seen that, in the presence of Cu^2+^ ions, the rhamnolipid aggregates were smaller and possessed higher AI values than those observed in the pure aqueous and KCl systems (Figure 3a,c). The smaller the size of the particle, the higher its velocity during electrophoresis [26].

**Figure 4:**
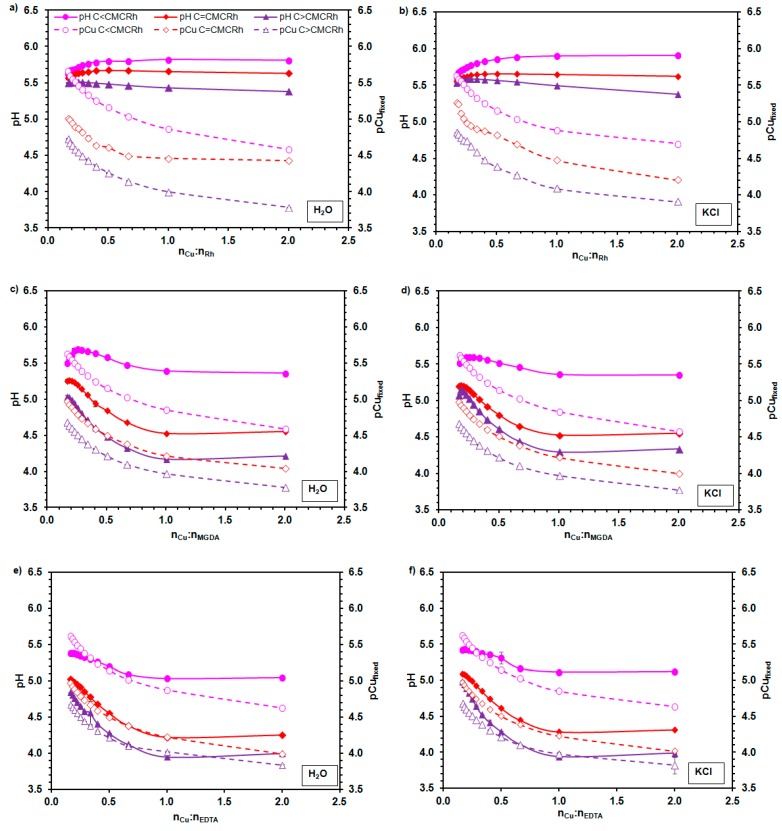
Dependence of pH and pCu_fixed_ (the negative logarithm of the concentration of Cu^2+^ ions fixed by chelating agent) on the Cu^2+^ to chelating agent molar ratio (n_Cu_:n_chelating agent_) in water (**a**,**c**,**e**) and KCl solution (**b**,**d**,**f**) at pH 5.5; (**a**,**b**) rhamnolipids (Rh); (**c**,**d**) methylglycinediacetic acid (MGDA); and (**e**,**f**) ethylenediaminetetraacetic acid (EDTA); CMC_Rh_ means the rhamnolipids’ critical micelle concentration.

**Figure 5:**
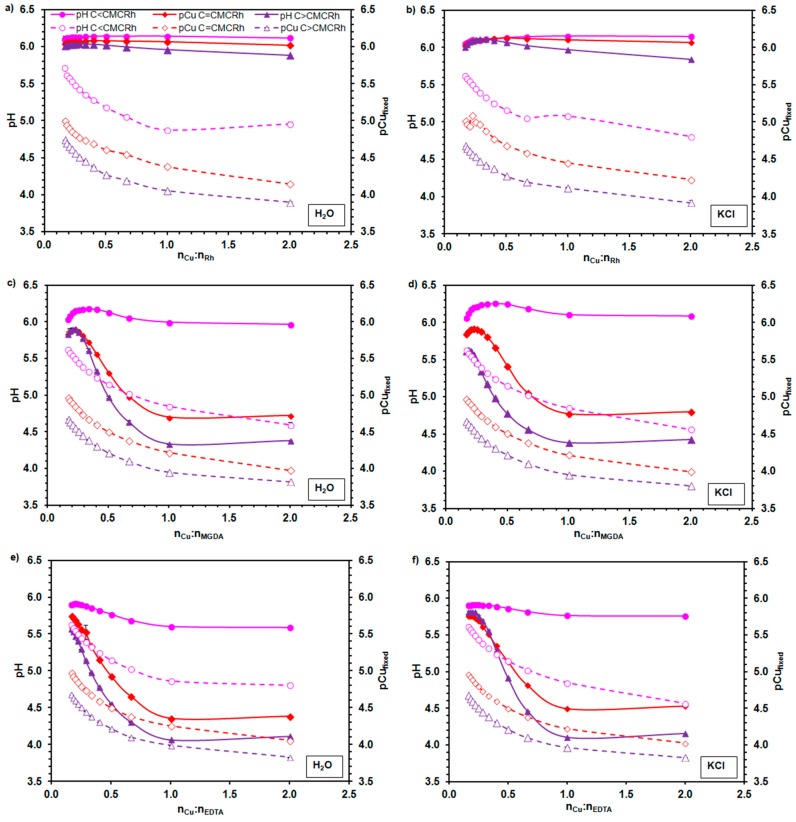
Dependence of pH and pCu_fixed_ (the negative logarithm of the concentration of Cu^2+^ ions fixed by chelating agent) on the Cu^2+^ to chelating agent molar ratio (n_Cu_:n_chelating agent_) in water (**a**,**c**,**e**) and KCl solution (**b**,**d**,**f**) at pH 6.0; (**a**,**b**) rhamnolipids (Rh); (**c**,**d**) methylglycinediacetic acid (MGDA); and (**e**,**f**) ethylenediaminetetraacetic acid (EDTA); CMC_Rh_ means the rhamnolipids’ critical micelle concentration.

The immobilization of Cu^2+^ ions by rhamnolipid molecules in premicellar and micellar solutions at pH 5.5 and 6.0 with and without KCl was determined by the potentiometric measurements. Both the pH and Cu^2+^ ions activity was monitored at the molar ratio of copper ions to rhamnolipids ranging from 16:100 to 2:1. The experiments were performed for the studied biosurfactants and two chelating agents (MGDA and EDTA). Three concentrations of the studied compounds were applied to check the chelating activity of rhamnolipids below the CMC, close to the CMC and in strongly aggregated form (over the CMC). The obtained results are shown in Figure 4 and Figure 5.

It can be seen, that for both the 5.5 and 6.0 pH values, as well as all the ionic strengths and chemical compounds used, the changes of pCu_fixed_ vs. the Cu^2+^ to the chelating agent molar ratio were similar. Approximately to the 1:1 the Cu^2+^ to chelating agent molar ratio, the fast increase of the amount of immobilized Cu^2+^ ions with the increasing total concentration of the Cu^2+^ added was observed. A further increase of the Cu^2+^ to chelating agent molar ratio did not cause such a significant increase of the Cu^2+^ immobilization. It should be marked that in this experiment the molar concentration of rhamnolipids refers to the number of moles of the mono- and dirhamnolipid pairs per liter of solution. Therefore, the results presented in Figure 4 and Figure 5 indicate that Cu^2+^ ions were complexed by two molecules of rhamnolipids at the 1:1 Cu^2+^ to chelating agent molar ratio. Moreover, it can be seen that the higher the concentration of chelating agents applied (i.e., below the CMC < about the CMC < over the CMC), the higher the copper fixation.

The shapes of dependences of pH on the Cu^2+^ to chelating agent molar ratio, which were obtained for both MGDA and EDTA, were different from those observed for the rhamnolipid solutions. For the MGDA and EDTA solutions the Cu^2+^ ions immobilization occurred simultaneously with the pH decrease. The CuSO_4_ hydrolysis did not cause such pH changes of the solutions, so the observed effect was connected with the process of cation chelation. In the case of rhamnolipids, at a concentration lower than the CMC, an increase of pH was noticed. Only for the highest concentrations of biosurfactants the slight decrease of pH with an increase of the Cu^2+^ to chelating agent molar ratio was observed. However, the recorded pH changes were not so significant, as in the case of MGDA and EDTA. It was found by Raza et al. [25] that in the 2 mM Sr^2+^ or Pb^2+^ ion solutions the CMC of rhamnolipids decrease twice with respect to that in the pure aqueous solution. Considering this, the smallest addition of CuSO_4_ to the premicellar solution of rhamnolipids initiated the aggregation process.

The pH of rhamnolipid solutions was established initially. The molecules in the premicellar solutions were more ionized that those in the aggregated state. The micellization and the cation immobilization, which occurred simultaneously, did not cause the increase of acidification in this case. In the micellar solutions of rhamnolipids at the concentration close to the CMC the copper fixation also did not lead to significant changes of pH. This suggests that the cations, which interacted with the free ions of rhamnolipids, intensified the aggregation process [25] and/or neutralized the initially ionized hydrophilic heads of monomers in the existing micelles. Only for the micellar solutions with the highest concentration of rhamnolipids did the partial exchange between Cu^2+^ and H^+^ ions occur, which led to a slight lowering of pH. The revealed properties of rhamnolipids can be important, e.g., when the pH of solutions should be constant during the cation chelation.

In the solutions of MGDA and EDTA in all studied cases the immobilization of Cu^2+^ led to the release of H^+^ ions.

An interaction of all the studied compounds with metal ions was characterized by the degree of Cu^2+^ immobilization and the stability constants of the formed complexes at 1:1 molar ratio of Cu^2+^ ions and chelating agent. The obtained results are summarized in Table 2 and Table 3.

The rhamnolipids revealed the best ability to immobilize copper ions in premicellar solution without KCl (over 90%) at all applied pH values. The obtained values correspond to those recorded for MGDA and EDTA. The competition between the Cu^2+^ and K^+^ ions lowered the copper fixation by rhamnolipids. An increase of concentration of the studied systems led to decrease of Cu^2+^ immobilization by all chelating agents. It should also be stressed that in the most aggregated state (C > CMC_Rh_), in the pure aqueous solution, the rhamnolipids showed Cu^2+^ immobilization at a similar level as MGDA and EDTA (about 70%). The worst results were obtained for rhamnolipids at the concentration close to CMC (50–60%). Both the MGDA and EDTA were not so sensitive to the presence of KCl as the rhamnolipids. The results obtained for MGDA and EDTA correspond to the literature data. The theoretical chemical simulations, which were performed at a 1:5 metal to chelating agent molar ratio, showed that at least 80% of Cu^2+^ should be complexed by these compounds at a pH ranging from about 1.5 to 10.0 [27].

The stability constants of the copper ions complexes with rhamnolipids, MGDA and EDTA decreased with the increase of the system’s concentration. The increase of pH and the addition of KCl lowered the values of the stability constants of the complexes formed by rhamnolipids. The stability of complexes formed by MGDA and EDTA was not so sensitive to pH and ionic strength. The obtained values correspond to these presented in the literature, i.e., 18.8 for EDTA [27] and 13.9 for MGDA [28] at 25 °C and 100 mM ionic strength. The values of stability constants which were calculated for the 1:1 mixture of mono- and dirhamnolipids are also comparable to those presented by Hogan et al. [29] for dirhamnolipids (i.e., 7.43–11.10).

The obtained results show that the mixture of mono- and dirhamnolipids is a good alternative to other ‘green’ (MGDA) or classically used (EDTA) chelating agents. However, its effectiveness depends on the system composition and pH conditions.

## 3. Materials and Methods

### 3.1. The Chemicals Used

The rhamnolipid R-95 (the 1:1 mixture of mono- and dirhamnolipids with the HPLC purity; the mean molecular weight of 577 g mol^−1^, p*K*_a_ = 5.6), methylglycinediacetic acid (MGDA; analytical grade, p*K*_a_ = 14.6 [28]), ethylenediaminetetraacetic acid (EDTA; analytical grade), potassium chloride (KCl), copper (II) sulfate (CuSO_4_; analytical grade), potassium hydroxide (KOH; analytical grade), and sulfuric acid (H_2_SO_4_) were purchased from Sigma Aldrich (Poznan, Poland) company.

Distilled filtrated (0.2 µm Whatman filters; GE Healthcare UK Ltd., Little Chalfont, UK) water with the electrolytic conductivity of 0.001 ms cm^–1^ at 20 ± 0.1 °C) was used for the solutions preparation.

### 3.2. Estimation of the Rhamnolipid Critical Micelle Concentration (CMC)

The CMC of the rhamnolipid mixtures was determined at pH 5.5 and 6.0, with ionic strengths of 0 and 20 mM at 20 °C. The rhamnolipid solutions with a concentration range from 0.022 mM (12.9 mg L^−1^) to 0.330 mM (189 mg L^−1^) were prepared by the addition of an appropriate volume of 0.690 mM (400 mg L^–1^) stock solution to water or 20 mM KCl solution. The pH of the rhamnolipid stock solution, as well as the water and KCl solution was established by the addition of 0.1 M KOH or 0.1 M H_2_SO_4_ to avoid the systems dilution. The ionic strength was adjusted by the use of 3 M KCl solution. The electrolytic conductivity (CDM210 conductivity meter with a CDC866T four–ring platinum electrode; Radiometer Analytical SAS, Lyon, France) and pH (TitraLab^®^ Titration Workstation D21T043 with a combined pH electrode; Radiometer Analytical SAS, Lyon, France) of rhamnolipid solutions were measured at 20 ± 1 °C in six replicates. The break points of the electrolytic conductivity and the pH vs. the rhamnolipid concentration curves were determined to estimate the CMC values.

### 3.3. Determination of the Particles Size and Electrophoretic Mobility in the Rhamnolipid Micellar Solutions

Micellar solutions of rhamnolipids (75.11 mg L^−1^, i.e., 0.130 mM and 150.21 mg L^−1^, i.e., 0.260 mM) at the ionic strength of 0 and 20 mM and pH 5.5 and 6.0 without and with the Cu^2+^ (1:1 molar ratio of Cu^2+^ to chelating agent) were characterized using the dynamic light scattering [30,31,32] and laser Doppler electrophoresis [33,34] methods. The measurements were carried out by means of a Zetasizer Nano ZS apparatus (Malvern Instruments Ltd., Malvern, UK) with the ‘size and zeta potential’ folded capillary cell in three repetitions at 20 °C. The viscosity (*η*) and the refractive index (*RI*) of the dispersant were those of pure water and 20 mM KCl solution (containing the appropriate amount of CuSO_4_), whereas *RI* of dispersed phase was that of the rhamnolipid’s saturated solution.

The mean hydrodynamic diameter d_h,app_ of surfactant aggregates was obtained using the 173° backscatter detection. Additionally, their mean diameter was determined using the forward detection (12.8°). Results of size measurements were used for the aggregation index (AI) calculation. This index is defined as a difference between the ratio of hydrodynamic diameters obtained at forward and backscatter and the number 1 [35]. The higher the value of AI, the more the system is aggregated [35].

The samples’ heterogeneity in respect to the particle sizes was described by the polydispersity index (PDI). The lower the PDI, the more homogeneous the system [30].

### 3.4. The Evaluation of Rhamnolipids’ Ability to Form Cu^2+^ Chelates

Solutions of rhamnolipids, MGDA and EDTA were prepared, respectively, at concentrations of 0.014 mM, 0.065 mM, and 0.130 mM in pure water and 20 mM KCl at pH 5.5 and 6.0. In the case of rhamnolipids this was the concentration of the monorhamnolipid-dirhamnolipid pair (i.e., the molecular weight of 1154 g mol^−1^) possessing two –COOH groups. Next, the 0.001 M CuSO_4_ at pH = 5.686 and the known ionic strength (0 or 20 mM KCl) was added to these solutions to obtain the 16:100, 18:100, 20:100, 22:100, 25:100, 28:100, 33:100, 40:100, 50:100, 67:100, 1:1, and 2:1 Cu^2+^ to chelating agent molar ratios. The activity of free Cu^2+^ ions in solution was measured potentiometrically (Cole–Parmer Replaceable Membrane ISE Probe–Copper; Cole Palmer, Vernon Hills, IL, USA) in three replicates. The copper electrode was calibrated using the series of CuSO_4_ solutions at the ionic strengths adjusted to those of the studied samples. The pH of the Cu^2+^ and chelating agent solutions was determined (TitraLab^®^ Titration Workstation D21T043 with a combined pH electrode; Radiometer Analytical SAS, Lyon, France) in six repetitions. To estimate the effect of CuSO_4_ hydrolysis on the solutions acidity and the Cu^2+^ ions content the pH of water and 20 mM KCl (pH 5.5 and 6.0) with the appropriate copper sulfate addition was measured.

All experiments were made at the temperature of 20 ± 1 °C.

Knowing the free Cu^2+^ ion concentration at a given CuSO_4_ content, the degree (%) of the metal binding was calculated at 1:1 metal ions to chelating agent molar ratio: *β* = [Cu^2+^_fixed_] × 100%/[CuSO_4_].

Considering that Cu^2+^ ions form with the rhamnolipids, MGDA and EDTA the complexes of ML_n_ type (M + nL ↔ ML_n_), the complexing constants (log*K*, where: *K* = [ML_n_]/([M_free_][L_free_]^n^) were calculated at the 1:1 metal ions to chelating agent molar ratio.

## 4. Conclusions

The 1:1 mono- and dirhamnolipid mixtures form aggregates at 0.094 mM and 0.115 mM in pure aqueous solutions at pH 5.5 and 6.0, respectively. In 20 mM KCl the critical micelle concentration of these biosurfactants was equal to 0.057 mM at pH 5.5, and 0.098 mM at pH 6.0. The aggregates possessed the negative surface charge.

In the presence of Cu^2+^ ions the diameter of rhamnolipid micelles ranged from 43 to 89 nm, dependently on the dispersing medium composition. The biosurfactant was in a strongly aggregated state (small-sized particles with the high values of the aggregation index).

Rhamnolipids revealed the best chelating activities in the premicellar solutions (over 90% of Cu^2+^ ions were bonded; the stability constant range: 10.75–11.10), a lower in the strongly aggregated state (over 60% of Cu^2+^ ions were bonded; the stability constant range: 8.07–8.38) and the lowest ones at the concentration close to the CMC (over 50% of Cu^2+^ ions were immobilized; the stability constant about 8.50). The presence of K^+^ ions lowered the Cu^2+^ binding by rhamnolipids without the significant effect on the complexes’ stability. The degree of metal binding as well as the stability constants of complexes formed by all chelants decreased with the increasing concentration of systems.

The immobilization of Cu^2+^ by rhamnolipids did not lead to a significant increase of acidification, opposite to the effect observed in the MGDA and EDTA solutions. That was probably connected with the surfactant molecules’ aggregation and structural reorganization in solutions.

The mixture of mono- and dirhamnolipids can be an alternative for the usually used synthetic chelating agents. However, its effectiveness depends on the system composition and pH conditions.

## Figures and Tables

**Table 1:** The critical micelle concentration (CMC) of 1:1 mono- and dirhamnolipid mixtures in water and 20 mM KCl solutions at pH 5.5 and 6.0.

pH	Analyzed Property	The CMC Determined in:			
		H_2_O		20 mM KCl	
		mg L^–1^	mM	mg L^–1^	mM
**5.5**	pH	42.46	0.074	32.14	0.056
	EC ^##^	66.39	0.115	34.04	0.059
		54.42 *	0.094 *	33.09 *	0.057 *
**6.0**	pH	69.64	0.112	58.28	0.101
	EC ^##^	68.09	0.118	55.39	0.096
		68.86 *	0.115 *	56.83 *	0.098 *

* The mean value of CMC; ^##^ EC means electrolytic conductivity.

**Table 2:** Amount of Cu^2+^ ions (%) immobilized by the analyzed compounds at a 1:1 molar ratio of Cu^2+^ to chelating agent in the different dispersing media, pH, and concentration applied.

pH	Substance	H_2_O			20 mM KCl		
		C < CMC_Rh_	C = CMC_Rh_	C > CMC_Rh_	C < CMC_Rh_	C = CMC_Rh_	C > CMC_Rh_
5.5	Rhamnolipids	95.82 ± 0.12	53.61 ± 1.37	77.63 ± 0.44	91.37 ± 0.51	51.72 ± 0.84	63.13 ± 1.34
	MGDA	98.24 ± 0.03	93.50 ± 0.03	83.08 ± 0.09	98.58 ± 0.01	93.86 ± 0.01	82.63 ± 0.17
	EDTA	92.37 ± 0.20	91.28 ± 0.28	73.34 ± 0.21	98.44 ± 0.03	91.54 ± 0.15	81.65 ± 6.60
6.0	Rhamnolipids	93.66 ± 0.18	64.06 ± 0.98	68.08 ± 0.58	58.08 ± 0.67	54.46 ± 0.47	58.83 ± 1.27
	MGDA	98.47 ± 0.03	92.83 ± 0.02	86.32 ± 0.09	98.58 ± 0.00	93.85 ± 0.01	85.96 ± 0.18
	EDTA	95.13 ± 0.34	85.76 ± 0.14	79.23 ± 0.20	98.58 ± 0.00	92.52 ± 0.08	82.78 ± 0.12

MGDA means methylglycinediacetic acid; EDTA means ethylenediaminetetraacetic acid; C means the chelating agent concentration; CMC_Rh_ is the rhamnolipids’ critical micelle concentration.

**Table 3:** The stability constants of complexes formed by Cu^2+^ ions and the analyzed compounds at a 1:1 molar ratio of Cu^2+^ to chelating agent in the different dispersing media, pH, and concentration applied.

pH	Substance	H_2_O			20 mM KCl		
		C < CMC_Rh_	C = CMC_Rh_	C > CMC_Rh_	C < CMC_Rh_	C = CMC_Rh_	C > CMC_Rh_
5.5	Rhamnolipids	11.10 ± 0.01	8.50 ± 0.02	8.38 ± 0.01	10.75 ± 0.03	8.48 ± 0.01	8.07 ± 0.03
	MGDA	14.86 ± 0.01	12.30 ± 0.00	10.93 ± 0.00	14.95 ± 0.00	12.33 ± 0.00	10.92 ± 0.00
	EDTA	18.22 ± 0.01	15.53 ± 0.01	13.75 ± 0.00	18.93 ± 0.01	15.57 ± 0.01	13.95 ± 0.15
6.0	Rhamnolipids	10.51 ± 0.01	8.29 ± 0.02	7.77 ± 0.01	9.50 ± 0.01	8.12 ± 0.01	7.60 ± 0.02
	MGDA	14.91 ± 0.03	12.24 ± 0.00	11.03 ± 0.00	14.94 ± 0.00	12.31 ± 0.00	11.02 ± 0.00
	EDTA	18.26 ± 0.01	15.12 ± 0.00	13.72 ± 0.00	18.81 ± 0.00	15.43 ± 0.00	13.82 ± 0.00

MGDA means methylglycinediacetic acid; EDTA means ethylenediaminetetraacetic acid; C means the chelating agent concentration; CMC_Rh_ is the rhamnolipids’ critical micelle concentration.
